# 

**Published:** 1882-02

**Authors:** 


					﻿Whole Number,
CGKXXV.
JMJARY, 1882.
Number 7,
Volume XXI.
THE
BUFFALO
Medical and Surgical Journal.
EDITORS:
THOS. I.OTHROP, M.
I*. W. VAN PEVMA, M. I).,
A. R. DAVIDSON, M.
LVCIEN HOWE, M.
HERMAN MYNTER, M. I».
BUZFZEVAEO':
Published by the Medical Journal Association.
Read the Notice of this Journal among the Advertisements.
Entered at the Post-Office at Buffalo, N. Y., as Second-Class Mail Matter.
				

